# Strength training and sarcopenia—a mandatory link: focus on MicroRNAs

**DOI:** 10.3389/fragi.2025.1554340

**Published:** 2025-07-30

**Authors:** Erika Cione, Diana Marisol Abrego-Guandique, Aldo Chiari, Roberto Cannataro

**Affiliations:** ^1^ Department of Pharmacy, Health and Nutritional Sciences, University of Calabria, Cosenza, Italy; ^2^ Galascreen Laboratories – University of Calabria, Cosenza, Italy; ^3^ Department of Health Sciences, University of Magna Graecia, Catanzaro, Italy; ^4^ Department of Promotion of Human Sciences and Quality of Life, San Raffaele Rome Telematic University, Rome, Italy; ^5^ Research Division, Dynamical Business and Science Society – DBSS Int. SAS, Bogotá, Colombia

**Keywords:** sarcopenia, strength training, miRNA, epigenetics, myomiR, resistance training

## Abstract

Over the last 20 years, increased life expectancy has been observed in men and women, resulting in a rise in the prevalence of diseases among the aging population. From this, sarcopenia has an estimated prevalence of 10%–16% of older people worldwide. Losing strength and muscle mass in the 65–70 age group represents a significant public health problem. In this review, we emphasize the essential importance of strength training in managing sarcopenia, highlighting the role of microRNAs, small nucleotides that were the subject of last year’s Nobel Prize in Physiology or Medicine. These microRNAs regulate protein synthesis and are present in all biological fluids. Some of them are expressed differently by subjects affected by sarcopenia (as happens in various forms of cancer or other diseases). Therefore, monitoring a specific signature of microRNAs can better clarify the etiopathology of sarcopenia, providing an early biomarker for sarcopenia (currently, there are some hypotheses, but none is well recognized), and even serve as the basis for the development of drugs.

## Graphical Abstract

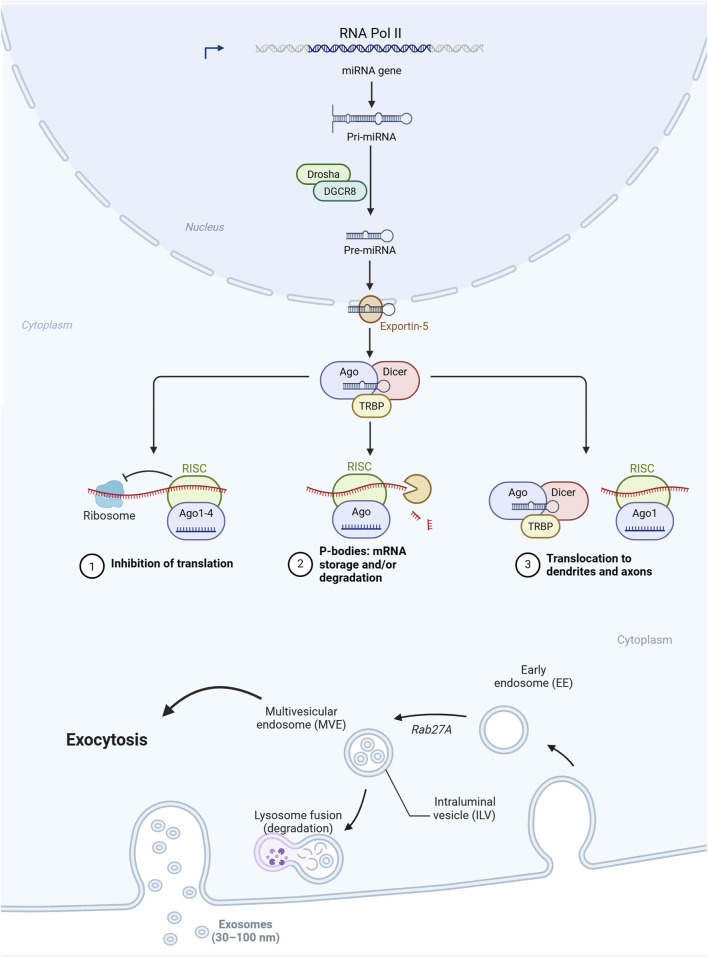

## Introduction

Over the last 20 years, particularly in Europe and the United States, increased life expectancy has been observed in both men and women, leading to a rise in the prevalence of diseases among the aging population. The healthcare system should ensure that this improvement occurs alongside an adequate quality of life (Sayer et al., 2024). One of the factors that can affect the quality of life with advancing age is sarcopenia, with an estimated prevalence of 10%–16% in older people worldwide (Sayer et al., 2024; Cannataro et al., 2021a). This condition manifests as decreased muscle mass and strength and has direct consequences for quality of life, including reduced mobility and difficulty maintaining an optimal posture. However, there are also indirect consequences, such as a greater incidence of falls and accidents, as well as a lower cognitive capacity, which appears to be increasingly linked to maintaining an optimal physical condition.

**FIGURE 1:**
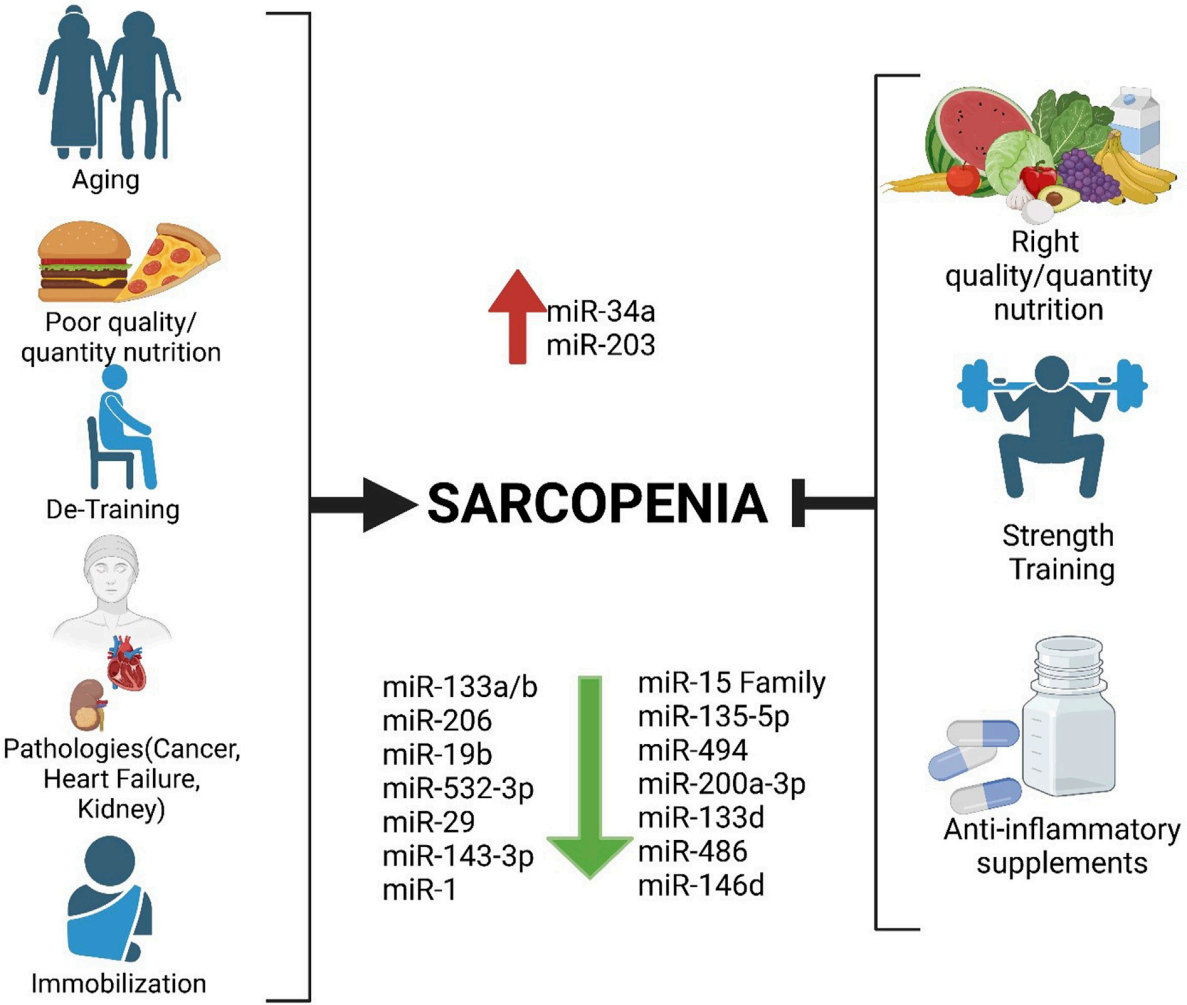
Factors favoring sarcopenia on the left and possible strategies to delay it by modulating microRNAs, including myomiR, on the right.

Previously, we demonstrated that sarcopenia management should not overlook a holistic approach, meaning that the condition should be addressed by various medical specialists (Cannataro et al., 2021a). Although its etiopathology is still not fully understood, lifestyle certainly has a significant impact on it. Lifestyle is intimately connected with nutrition and physical activity, which in turn regulate epigenetic mechanisms. In this view, it is necessary to ensure that sarcopenia patients ingest a sufficient intake of polyphenols, which can be achieved through a diet that incorporates healthy habits, such as a good amount of vegetables and fruits, as recommended in Mediterranean diet guidelines (Cannataro et al., 2021b). When necessary, supplementation with omega-3 fatty acids for their anti-inflammatory properties should be recommended (Cannataro et al., 2024), as well as supplementation with micronutrients, such as creatine or beta-carotene, to improve cognitive functions (Elechi et al., 2024; Abrego-Guandique et al., 2023). It is well known that nutrition and physical activity influence the epigenome, on which microRNAs (miRNAs) have a role (Plaza-Diaz et al., 2022; Abrego-Guandiq et al., 2025). In this literature review, we will focus on the importance of physical activity, particularly strength training exercises, which could be essential in treating sarcopenia—a condition currently without a known pharmacological cure. To this as a methodical strategy, we searched on Scopus and PubMed, using the keywords “sarcopenia,” “miR,” “microRNA,” “myomiR,” and “strength training” during July 2024, selecting 21 manuscripts that identified 16 miRNAs that are altered in sarcopenia and also influenced by lifestyle, and 12 miRNAs that are influenced by physical exercise in sarcopenic condition (Figure 1). After analyzing the pathophysiology of sarcopenia and explaining the biogenesis of miRNAs, with a focus on those specific to a skeletal muscle called myomiR, we will consider those related to i) sarcopenia, ii) strength training, and iii) sarcopenia and physical activity in older people. Our goal is to provide a scenario of the current situation and propose some miRNAs as possible candidates for the diagnosis, prognosis, and treatment of sarcopenia.

## Sarcopenia: definition, diagnosis, and pathophysiology

Sarcopenia is characterized by a loss of muscle mass, strength, and function, leading to physical disability and an increased chance of fatality, worsening the overall quality of life (Sayer et al., 2024; Cannataro et al., 2021a). It primarily occurs in older people due to the aging process. Muscle mass tends to decrease throughout life. The physiological decrease in muscle mass is approximately 1% per year after the age of 30, with a significant acceleration after the age of 70. This physiological process, as well as the lack of regular and organized physical activity, contributes to the prevalence of sarcopenia, which is often not diagnosed and certainly not diagnosed early, and is estimated to be at 10%–16% before the age of 70. This percentage increases at least three-fold after the age of 80. Sarcopenia is often associated with muscle atrophy, defined as the shrinking of muscle fibers, accompanied by loss of function and a decrease in cellular organelles, particularly the mitochondria. Another phenomenon of great importance is an inflammatory process that tends to exacerbate the condition (Supriya et al., 2021; Dalle et al., 2017). In addition, the senescence of muscle satellite cells is observed in sarcopenia, where the replacement of damaged cells is impaired, resulting in reduced functionality. On the other hand, it is exciting to note that the response to physical exercise, especially strength exercise, is comparable between older and younger subjects, with the difference that young subjects require shorter recovery times between one workout and another (Fritzen et al., 2020).

Sarcopenia diagnosis is difficult, and the criteria were clinically described by the European Working Group on Sarcopenia in Older People 2 (EWGSOP2) and the Extended Group for EWGSOP2 (Cruz-Jentoft et al., 2019). Three parameters are used to diagnose it: i) grip and chair stand tests; ii) skeletal muscle mass evaluation; iii) gait speed test, short physical performance battery (SPPB), and the timed-up-and-go test (TUG). The grip and chair stand tests are the first and most important tests, followed by the muscular strength evaluation. The skeletal muscle mass evaluation is a challenge. Advice for this scope is to use dual-energy X-ray absorptiometry (DXA), computed tomography (CT), or magnetic resonance (MR).

Additionally, bioelectrical impedance analysis (BIA) provides valuable feedback and is easier to execute than DXA, CT, and MR, making it a more cost-effective option. It can also provide indications of the phase angle related to the overall health status. Finally, physical performance was evaluated using gait speed tests, two dedicated tests (SPPB), and the timed-up-and-go test (TUG). Regrettably, each method has its limits. For example, the grip test depends on the instrument used and the general conditions of the subject. Having a “history” of the measurements would be helpful, but it is often impossible. DXA, CT, and MR are reliable but expensive and not easily applied, especially for follow-up purposes. BIA is easy to use and relatively inexpensive; however, the equations developed for older people require further validation (Campa et al., 2023). As a new tool, ultrasound-based technology could represent a valid alternative, as it is non-invasive, inexpensive, and, if well-developed, could provide suitable indications (Annunziata et al., 2025).

The pathophysiology of sarcopenia is multifactorial, even at the molecular level, but it is primarily attributed to impaired muscle homeostasis. Skeletal myogenesis triggers satellite cell differentiation, with consequent fusion of new myoblasts into mature myotubes (Sousa-Victor et al., 2022; Sartori et al., 2021). Myogenic regulatory factors (MRFs) are a class of mediators that supervise differentiation and growth: myogenic differentiation D (MyoD), myogenic factor 5 (Myf5), myogenin, and myogenic regulatory factor type 4 (MRF4) (Schiaffino et al., 2013; Dewi et al., 2023) contribute to the differentiation. Usually, satellite cells are in a quiescent state and show increased expression of the paired box transcription factor type 7 (Pax7). During myogenic differentiation, the expression of Pax7 protein is suppressed, and at the same time, MRF4 and myogenin expressions are upregulated, resulting in myotube maturation (Gao et al., 2018). Even the role of Pax7 could be revised following the report of Jackson et al. (2012), which shows that satellite cells are not required for muscle regrowth or atrophy, even in mice. A pivotal role could be assigned to MRFs, which are essential for muscle development and satellite maintenance. It could be one of the primary mechanisms in the etiopathogenesis of sarcopenia, leading to a quantitative decrease in satellite cells themselves, as reported in human and murine models (Crossland et al., 2013; Roberts et al., 2023). Molecular and transductional pathways involved in sarcopenia include transforming growth factor beta (TGF-β), bone morphogenetic protein (BMP), and insulin-like growth factor 1 (IGF-1) (Jackson et al., 2012; Crossland et al., 2013; Roberts et al., 2023; Lan et al., 2024). The TGF-β signaling pathway inhibits skeletal muscle growth, leading to atrophy (Gao et al., 2018; Jackson et al., 2012). TGF-β type 1, activin A/B, myostatin (also known as growth differentiation factor 8, GDF8), and GDF11 are well-established negative regulators of muscle development and growth (Jackson et al., 2012; Lan et al., 2024 Machelak et al., 2023). Consequent to TGF-β binding to its receptor, Smad 2/3 is phosphorylated, inducing the translocation of forkhead box O (FOXO) transcription factors. These transcription factors trigger protein degradation and consequent muscle deterioration (Martins et al., 2016). The phosphorylated Smad 2/3 also interacts with the cyclin-dependent kinase (CDK) inhibitors p21 and p27, inducing skeletal muscle senescence (Englund et al., 2023).

IGF-1 plays a fundamental role in the skeletal muscle physiology (Schiaffino et al., 2013; Yoshida and Delafontaine, 2020); it acts via a tyrosine kinase receptor, the second messenger of insulin receptor substrate (IRS) types 1 and 2. IRS-1 and -2 lead to the activation of the phosphoinositol 3-kinase (PI3K)-Akt (Weeks et al., 2017) axis. The activated Akt promotes muscle mass and strength by inhibiting FOXO and activating mTOR, a potent activator of protein synthesis (Ersahin et al., 2015). The muscular, autocrine isoform of the IGF-1 called MGF, which is secreted by muscle in response to exercise, particularly strength training, is of great interest (Ahtiainen et al., 2011). Of note, decreased IGF-1 expression is associated with aging, and even sarcopenia is related to an impaired IGF-1 signaling pathway (Cannataro et al., 2021a; Crossland et al., 2013; Goldspink, 2006).

## Biochemistry of microRNA and their cargos in macromolecules

In 2024, the Nobel Prize was awarded to the two scientists Ambros V. and Ruvkun G. (Lee et al., 1993; Wightman et al., 1993), who, 32 years ago, first discovered microRNAs (miRNAs). Since then, research in this field has multiplied, making thousands of sequences known.

MiRNAs are RNA sequences that count 20–25 nucleotides. Most originate from the intronic gene regions, and a few originate from the exonic ones. Sometimes, they are transcribed in longer sequences called clusters. The prevalent biogenetic pathway is identified, where a pre-miRNA with the typical hairpin shape is transcribed via an RNA-binding protein, DiGeorge Syndrome Critical Region 8 (DGCR8), and a ribonuclease III enzyme, Drosha. The pre-miRNA is exported into the cytoplasm via a specific exportin and then processed by the RNase III endonuclease, Dicer, which removes the terminal part, resulting in a duplex miRNA. Both are loaded into the argonaute (AGO) family of proteins, which acts as a sort of sequence controller, activating, if necessary, the degradation of the miRNA. The biochemical mechanism of miRNAs is typically epigenetic and almost always repressive. The binding to this latter sometimes results in the induction of transcription, but this last biochemical mechanism still needs to be well consolidated. Hence, the interaction with the complementary sequence of the RNA region described is referred to as miRNA response elements (MREs). In animals and, in particular, in mammals, the pairing is rarely complete, and almost all MREs contain an unpaired central part. This means that a miRNA can pair with more than one nucleotide sequence. It has been postulated that miRISC could regulate the chromatin state and, therefore, control transcription; however, this remains to be confirmed and elucidated. The biochemical activity of miRNA is a dynamic process that allows it to buffer gene expression. miRNAs are found in all cellular compartments. The network orchestrated by miRNAs is very complex because an RNA sequence can contain more than one MRE.

The presence and consequent biochemical action of miRNAs can also be influenced by the intracellular pool, the localization, and the release of cargo structures, as also revealed by long-non-coding RNAs (lncRNAs). These non-coding RNAs have longer sequences than miRNAs and act as cargo for miRNAs, regulating their action after they have been synthesized and released into the cytoplasm. Probably one of the most interesting characteristics of miRNAs is the possibility of circulating; in fact, they have been found in all body fluids: blood and serum (Ryan and Atreya, 2011), milk (Ahlberg et al., 2023), saliva (Gao et al., 2018), urine (Záveský and Slanař, 2023), lymph (Yusof et al., 2021), and cerebrospinal fluid (Cogswell et al., 2008).

Unlike messenger RNA (mRNA), which is usually rapidly degraded, miRNAs are stable even in non-physiological temperature and pH conditions and can also be frozen without being altered (Kupec et al., 2022), most likely because they circulate in exosomes, the minor subpopulation of extracellular vesicles (EVs), or are complexed with proteins such as AGO (Turchinovich et al., 2011; Arroyo et al., 2011), but also high- and low-density lipoproteins (HDL and LDL), and nucleophosmin 1 (NPM1) (Michell and Vickers, 2016). The complex formed by exosomes, EVs, or proteins protects miRNAs from degradation, allowing them to remain stable for extended periods. Initially, it was thought that miRNAs were a waste product of cells and, therefore, present in biological fluids as part of apoptotic bodies or, in any case, without a biological function. It is now well established that miRNAs have a communication function between cells. For example, Guo et al. (2023) highlighted how there is a dense crosstalk between adipose and skeletal muscle tissue through cytokines and miRNAs carried by exosomes. Specifically, it has been seen that there are specific receptors for the internalization of EVs. Similarly, miRNAs complexed with proteins can exploit the relative receptors, such as those of HDL or LDL. This feature is crucial when considering miRNAs as biomarkers as it allows for easy transport of samples, even refrigerated or frozen. Therefore, the presence of EVs or the formation of complexes with lipoproteins protects miRNAs from the action of degrading enzymes but also from temperature and pH excursions. It also opens new scenarios from a physiological point of view, as it links the transport of miRNAs to specific receptors such as those of lipoproteins.

## Tissue microRNAs and myomiRs

The first discovery of impaired miRNAs was related to their release or up/downregulation in the tumor tissue. The idea was to search for specific miRNA signatures expressed in an aberrant manner by the tumor microenvironment and released in the blood circulation to be able to have an early cancer diagnosis. To this, the term liquid biopsy was coined (Raza et al., 2022; Lin et al., 2022). In general, any pathological condition could be linked to an altered expression of one or more miRNAs. It also opens the possibility of obtaining biomarkers not only for the early diagnosis of tumors but also for pathologies whose diagnosis is currently only clinical. For example, our group has recently identified 13 miRNAs profiled from adipose tissue biopsy in subjects affected by lipedema (Cione et al., 2024).

Additionally, research is being conducted to identify characteristic “signatures” of pathologies, with attention to the impact of diet or lifestyle (Colpaert and Calore, 2019; DeLucas et al., 2024; Slota and Booth, 2019). The latest frontier is the use of miRNAs as drugs or the use of synthetic miRNAs or complementary sequences that can block the action of miRNAs, called antagomiRs (Cannataro and Cione, 2023; Pagoni et al., 2023). Although promising, this idea also has some important limitations: reaching the target tissue, as the uptake by cells is not yet well understood. As repeatedly underlined, the action of miRNAs is almost always pleiotropic. Therefore, the specificity and the absence of side effects are not guaranteed. Like all other tissues, the skeletal muscle expresses miRNAs. They are particularly expressed by the skeletal muscle and are related to mechanisms vital for the functioning of the muscle itself; however, they are not exclusively produced by skeletal muscle. Therefore, they can be considered a specific characteristic, but attention must be paid to regulation by other tissues or organs. It has been reported that they are closely linked to skeletal muscle in response to physical exercise and to pathologies related to the musculoskeletal system (Pagoni et al., 2023; Noh et al., 2025; An and Wang, 2021; Qin et al., 2023). For example, miR‐214 (Noh et al., 2025) shows an altered expression in amyotrophic lateral sclerosis (ALS) patients, and it could be considered a prognostic biomarker. In sarcopenia, An and Wang (2021) identified miR-1245a as a potential hallmark, being differently expressed in sarcopenia subjects. The meaning needs to be better elucidated, but it appears linked to various kinase enzymes. In addition, many miRNAs (not all are found in body fluids; some remain confined in the cells that synthesize them) act as messengers, with action on various other tissues and organs: heart, adipose organs, bones, and the central nervous system (CNS) (Fulzele et al., 2019). In particular, Qin et al. (2023) highlighted the inhibitory action of muscle-secreted miR-146a-5p on adipogenesis, which could be useful in sarcopenia treatment. Fulzele et al. (2019) showed that miR-27a and -34a secreted by muscle are internalized by osteoblasts, thereby regulating osteogenesis. Unfortunately, as already underlined, miRNAs appear to be influenced by various factors, and it is difficult to correlate a single miRNA univocally to a biochemical event. In our previous work, we showed that a ketogenic diet strongly influences miRNA expression, with a slight but significant difference between men and women (Cannataro et al., 2019). In his review, Mallett shows that miRNAs follow a different pattern of expression in endurance or strength training, reflecting the overall epigenetic milieu, due to the different demands of training stimuli.

## Strength training: focus on older people and sarcopenia

Strength training (ST) is a type of physical exercise in which the muscular system is subjected to external resistance, which can be represented by weights, body weight alone, or tools such as elastic bands (Cannataro et al., 2022; Garber et al., 2011). If the organism is put in the right conditions, the phenomenon of super-compensation is generated; immediately after physical exercise, there is a decrease in performance, defined as over-reaching (OR) (Bell et al., 2020). If the right amount of rest and nutrients follow, the 4R scheme is supplied (Bonilla et al., 2020). The OR is functional for a performance improvement, specifically muscle strength and possibly muscle hypertrophy. If the stimulus is too high and/or the rest and refeeding are insufficient, it will be a non-functional OR with loss of function until the possibility of reaching the overtraining syndrome (Bell et al., 2020). In the past, ST was relegated to only a few sports; now, practically all sports include more or less prolonged periods of ST, even those that are purely endurance-based, such as running or cycling (Bonilla et al., 2020). Similarly, even in the general population, ST is increasingly considered in guidelines relating to general wellbeing (Garber et al., 2011; Meeusen et al., 2013; Riebe et al., 2015). In our previous work, we have underlined the fundamental importance of strength training in managing sarcopenia (Cannataro et al., 2022). Strength training is fundamental in aging and the treatment/prevention of sarcopenia. The benefits are related to the maintenance of the strength of skeletal muscle, with an important impact on the quality of life and on the reduction of the risk of falls; bone health is positively affected by ST, and even cognitive function, as shown by Cheng et al. (2022), is improved. It is important to remember that strength training with heavy loads can be applied in the case of osteoarticular pathologies and in people over 60 years of age. For example, we managed a case of knee osteoarthritis, obtaining a remission of the problem and a notable improvement in the quality of life in a 70-year-old woman, by performing a powerlifting-style training. At the end of the 2-year course, the patient obtained a 1-repetition maximum (1RM) in the deadlift of 105 kg (Malorgio et al., 2021). Obviously, we do not want to say that this can be applied to all situations, but it is undoubtedly an option to consider: evaluating the initial state, operating a slow and controlled progression, and with the supervision of dedicated and skilled personnel. Another option that has had a good response during the lockdown due to COVID-19 is home-based training, which could be valid in the case of subjects with poor mobility, but we think that live supervised training has a greater value.

As highlighted in our previous work, the methods can be the classic weight lifting ones, but also different ones such as those using sand-bags, elastic bands, or simply body weight, which for older subjects can represent an adequate load for improving strength (Cannataro et al., 2022). Chen et al. (2018) have shown that 8 weeks of training with kettlebells (a weight with a particular shaped grip, with dedicated training schemes) has produced improvements in the indices related to sarcopenia and to low-grade inflammation. Some of the results were maintained even after 4 weeks of de-training. The report by Marzuca-Nassr et al. (2023), where adults aged 65–70 and 85 or more obtained the same results in terms of strength, confirming that this type of training can be applied at any age, is interesting. In this case, the training was based on isotonic machines with 10 repetitions and a percentage of 1RM between 65% and 80%. Otsuka et al. (2022) noted that, evaluating both the quantity and the quality of the muscles (through DXA, MR, BIA, and strength tests), 40% of the 1RM is sufficient to improve the quantity, but the quality and therefore the strength require a higher percentage, that is, at least 60%. Another study by Chen et al. (2017) showed that only ST, when compared to endurance alone or mixed training, produced an increase in IGF-1.

In general, various works report an improvement following the ST. Unfortunately, the parameters used are nonuniform. We agree with the conclusion that many authors propose, for which it is not possible to provide a standard of both type and intensity of training, for we underline once again the need for careful and personalized supervision (Cannataro et al., 2022; Morcillo-Losa et al., 2024; Lu et al., 2021; Zhao et al., 2022). Finally, it should be emphasized that combining strength training with some nutrients, especially creatine, could synergistically affect muscle condition (Dos Santos et al., 2021) and indirectly promote cognitive function (Elechi et al., 2024), which is indispensable for exercise execution. Specifically, there are not many studies on creatine and ST, but there are some interesting results. Candow et al. (2015) show how a creatine supplementation (0.1 g × kg) for 32 weeks combined with ST showed a more marked improvement than ST alone in the 1RM of leg press and chest press and an improvement in body composition assessed with DXA.

## Sarcopenia: microRNA and physical exercise

**FIGURE 2:**
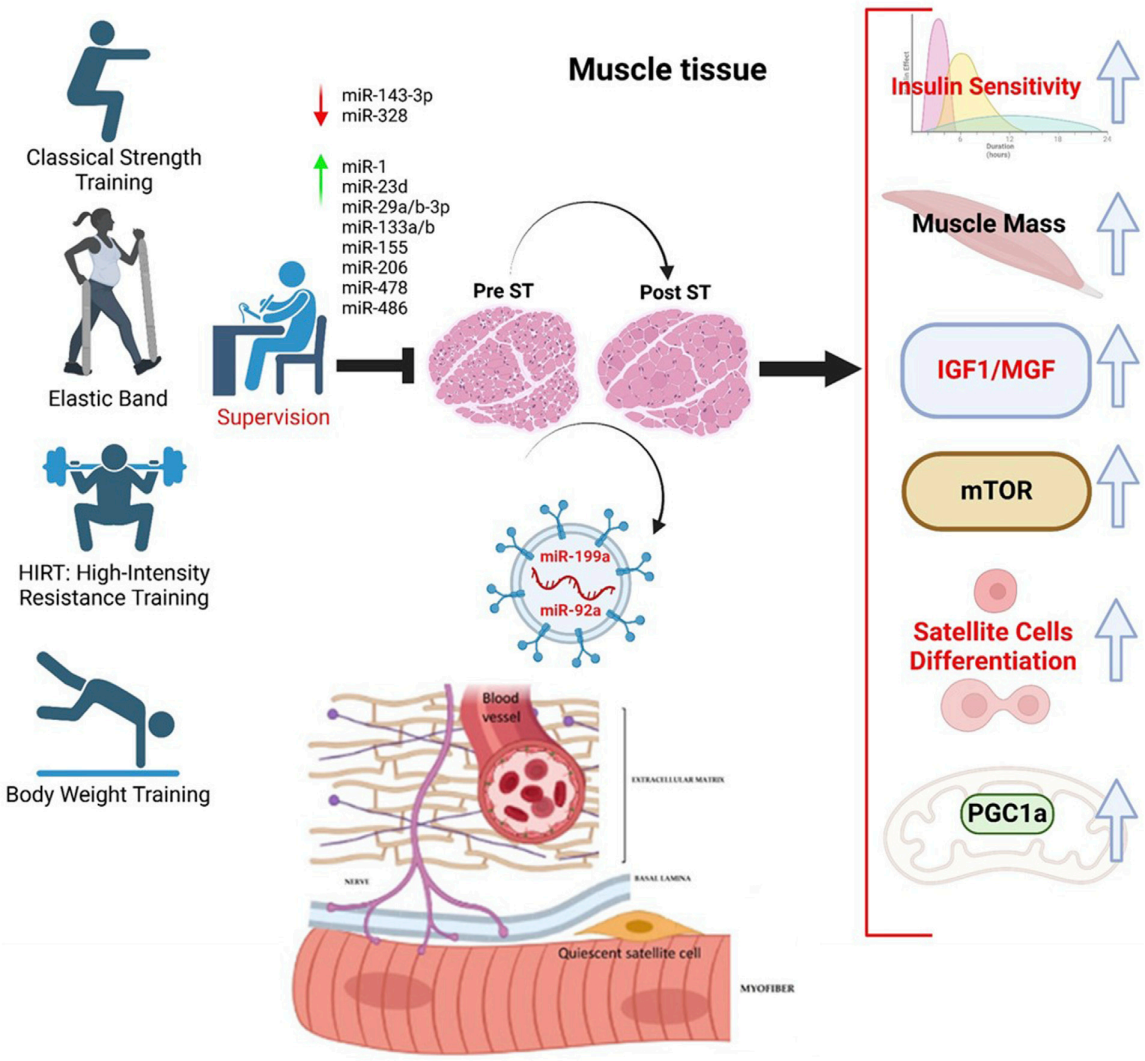
MicroRNAs modulated during supervised strength training (ST) and their action on related biochemical pathways favoring satellite cell differentiation. The miRNAs considered are all circulating, making them possible candidates as biomarkers. We have selected miRNAs in response to ST.

Unfortunately, the scientific works conducted with humans are not uniform. The primary concerns are linked to the age groups, the evaluation of the results obtained, and the types of training practiced. Nevertheless, the benefit of ST was highlighted in the variation in miRNAs. In the work of Xhuti et al. (2023), the expression of some miRNAs in older and younger subjects was first compared, and then the older subjects were subjected to training with elastic bands with progressive loads for 12 weeks. It is exciting to note that physical exercise affected some EV miRNA cargos, reflected in tissue muscle biopsies, for miR-1, -206, and -133a, with no action on miR-34a (Chen et al., 2023). Notably, the levels of miR-199a and -92a decreased in muscle tissue and increased in circulating extracellular vesicles (EVs), highlighting a biochemical action specifically linked to IGF1 and the differentiation of satellite cells (Figure 2).

These two phenomena are decisive in the condition of sarcopenia. Similarly, Agostini et al. (2021) monitored seven miRNAs in sarcopenic patients compared to healthy controls, noting some differences. After rehabilitation, the sarcopenia condition was more severe, and only miR-451a was decreased. This miRNA could make it a potential biomarker of the success of physical activity plans applied to sarcopenia. Although not tested on humans, a group of miRNAs related to physical exercise, particularly strength, is characteristic of sarcopenia. This emerges both from animal models and from studies with humans. For example, Millet et al. (2024) highlighted in 18 sarcopenic subjects, compared to as many healthy controls, a downregulation of miR-133a-3p and -200a-3p. In particular, the first is a myomiR, thus confirming the relationship with this group of miRNAs. The study by Xu et al. (2022) correlates another myomiR, miR-1, with the incidence of cardiovascular diseases in sarcopenic subjects, highlighting its downregulation, which is well correlated with a reduction in muscle mass. A downregulation of miR-532-3p was highlighted in the study by Chen et al. (2020) (Table 1).

**TABLE 1:** MyomiR, pathway, and action on sarcopenia.

miRNA	Maior pathway involved	Action on sarcopenia
−1	IGF-1, MyoD, and myostatin	Impaired muscle regeneration; improvement of bone/adipose tissue/heart
−15^a^	IGF-1 and IRS-1	Positive response to training
−23a	TGFβ	Inhibition of cell differentiation; muscle hypertrophy
−29	IGF-1	Tissue remodeling, cell cycle regulation, and antioxidant status
−34a	Sirt1	Muscle cells and bone marrow senescence
−92a	PTEN/AKT	Cell cycle progression
−133a/b	IGFR, UCP, and PGC1α	Impaired myoblast differentiation and fiber shift; diminished mitochondrial activity
−135-5p	MRF	Myogenesis impairment
−143-3p	IGF-1	Impaired regeneration, increased cell senescence
−144-5p	LC3II/LC3I and Beclin-1	Myoblast senescence, myogenic differentiation, and autophagy
−146a	NFkB	Inflammation, oxidative state
−199a	WNT2, NFkB	Regulation of cell cycle, cytoskeleton remodeling, and inflammation
200a-3p	TFG-β2/SMAD, CPK	Impaired myoblast proliferation and differentiation
−206	HDAC4, PAX7, MyoD	Impaired muscle regeneration, oxidative stress management
−431	TGFβ	Inhibition of cell differentiation, muscle hypertrophy
−532-3p	BAK1	Apoptosis induction, inflammation

^a^ Family.

This miRNA is associated with the inflammatory state, particularly with NF-κB. It is fascinating, as inflammation is one of the key points for managing sarcopenia. In a literature analysis, Ghafouri-Fard et al. (2023) reported on a group of miRNAs, mostly downregulated, in animal models of sarcopenia. They reported that miR-193b linked to the mTOR pathway, miR-223 linked to IGF1 and MyoD, miR-34a linked to bone marrow senescence, miR-320 linked to myoblast differentiation, miR-378 linked to the IGF1 receptor, let-7g-5p linked to inflammation via TNF-alpha and IL-6, and, lastly, miR-322 and -503 linked to eukaryotic initiation factors (eIFs). Finally, Lee and Kang (2022) clustered miRNAs into three pathways: i) IGF1 pathway blockade with miR-29, -143-3p, and the -15 family; ii) MRF-related miR-135-5p; and iii) TGF-beta with miR-431 and -23a. The data are summarized in Table 1. The authors also emphasize lncRNAs that act as a repository/regulator of the action of the miRNAs themselves. For example, in this case, LnclRS1 regulates the action of the miR-15 family by then acting on IRS-1.

## Future directions

Considering these results as a whole, some conclusions can be drawn, even if they are not definitive. They are nevertheless useful to direct the current management of sarcopenia and to structure future studies:

❖ Sarcopenia is a multifactorial pathological condition; training, in general, and in particular strength training, is a point that cannot be overlooked.
❖ Strength training can be considered in all its variants; however, it must be adapted to the individual situation.
❖ Kinesiologists and sports technicians must personalize training to make it both effective and safe.
❖ The analysis of miRNAs can be a valid support. Monitoring the miRNAs altered in sarcopenia, mainly linked to three pathways: i) IGF-1, ii) Akt/mTOR, and iii) PGC1-alpha, could be considered, as well as some related to inflammation, another characteristic of sarcopenia.
❖ Not all miRNAs found are positively influenced by training, but this could depend on the type of training applied and/or the subject rather than some intrinsic characteristics of the miRNAs themselves.
❖ The identification of specific signatures of miRNAs, including myomiRs, could be validated in a large cohort.
❖ Future therapy could be represented by synthetic miRNAs capable of carrying out a direct or indirect action by blocking or delaying mRNA translation into protein linked to the suppression of satellite cell differentiation.

The miRNAs could represent a handy diagnostic and drug-like tool. However, some conditions require greater attention. The first point is the potential of each miRNA to influence multiple metabolic pathways because the pairing of pathway and mRNA is not complete. It is necessary to analyze as best as possible all the possible variables that can influence the synthesis and action of the miRNA itself. For example, lifestyle, diet, or other concomitant pathologies can have a strong influence. Another important point is the standardization of analysis methods and sample collection. Although stable in EVs, miRNAs can be degraded. Furthermore, when considering plasma samples, we must be careful not to cause hemolysis as this would alter the sample’s miRNA content. Some trials are underway with synthetic miRNAs. However, as reported in the review by Seyhan (2024), phase I and II studies are underway. Therefore, without a result that can be used for pharmacological use, the major problem is to convey the miRNA to the target tissue, without having side effects on other tissues. The preferred route is the liposomal one, even if various alternatives are being evaluated, such as viral or bacterial vectors, gold-based particles, carbon, or synthetic or natural polymers. In the future, it would be beneficial to structure trials that include the analysis of miRNAs in response to ST in conditions of sarcopenia. However, the ideal would be to have a concurrent evaluation of the primary outcomes in the evaluation of strength (1RM, gait speed, handgrip test. IMAT), quality of life in general (QoL test and similar), omics studies, and expression of cytokines, possibly using a wearable device, to evaluate the adherence and effectiveness of training programs. Another point to consider is the possible difference in output in pre-sarcopenia, overt sarcopenia, and possibly primary and secondary sarcopenia. Specifically, regarding miRNAs, a better characterization of the panels is necessary, making sure that there are no other factors that influence the expression and action. In addition, the methods of extraction and purification of EVs and analysis of miRNAs should be standardized to develop a routine diagnostic method.

## Conclusion

Currently, many mechanisms are either hypothetical or supported by few studies. Therefore, miRNAs could represent a tool to better elucidate the pathogenesis of sarcopenia. In the same way, miRNAs could represent a biomarker or even the basis for a drug. However, there are numerous points to evaluate carefully and in a more in-depth manner: the specificity of miRNAs, the delivery, and the possible side effects. There is still much to investigate. There is a need for large trials involving various investigation methods. For different and larger populations, the connection between the various outcomes, already used routinely, such as the grip test and the DXA or not, such as omics or cytokine analysis; and the use of wearable devices to support validations should not be overlooked. A unanimous consensus would also be helpful to program validation of miRNAs as biomarkers through standardized work protocols.
